# All-Inside Arthroscopic Anatomic Anterior Talofibular Ligament Repair for Chronic Lateral Ankle Instability and Injury at the Talar Attachment: Surgical Technique

**DOI:** 10.1016/j.eats.2024.103098

**Published:** 2024-06-24

**Authors:** Nanami Ueda, Yasutaka Murahashi, Katsunori Takahashi, Yuta Mori, Kota Watanabe, Makoto Emori, Atsushi Teramoto

**Affiliations:** a Department of Orthopaedic Surgery, Sapporo Medical University School of Medicine, Sapporo, Japan; b Department of Physical Therapy, Sapporo Medical University School of Health Sciences, Sapporo, Japan

## Abstract

Recently, arthroscopic anterior talofibular ligament (ATFL) repair has become popular, and favorable outcomes have been reported. In general, ATFL injuries are often caused by fibular attachment, and there are no reports of arthroscopic ligament repair of talar attachment injuries. We present a surgical technique for arthroscopic ligament repair via the anterolateral portal, accessory anterolateral portal, and far accessory anterolateral portal for ATFL injuries on the talar side. Ligament plication is performed using a suture anchor at the talar footprint of the ATFL after the small bone fragments are removed under arthroscopy. Arthroscopic surgery may lead to less postoperative swelling and pain than open surgery, allowing for early exercise and return to activity.

Lateral ankle sprains are the most common type of ankle sprain, of which approximately three-quarters are anterior talofibular ligament (ATFL) injuries.^1^ The ATFL runs nearly horizontally when the ankle is in a neutral position but runs nearly parallel to the long axis of the leg during plantar flexion. Therefore, ATFL injury is likely to occur when the ankle is plantar flexed and inverted.^2^^,^^3^ ATFL injuries are classified as ligament-only and avulsion fractures.^4^ Most injuries occur on the fibular side, and there are few reports of injuries on the talar side.

Treatment of ATFL injuries is usually conservative, such as cast fixation, weight reduction, and application of functional braces in new cases. Surgical treatment may be considered when ankle instability or pain persists in chronic cases even after conservative treatment.

Since Broström^5^ first reported open ligament repair in 1966, good results have been reported in many cases, and it has become the gold-standard surgical technique. Recently, arthroscopic ligament repair has been reported to yield good results.^6^ The potential advantages of arthroscopic surgery include less invasiveness, the ability to perform intra-articular lesion treatment, and minimization of postoperative pain and scarring, as well as faster recovery, making it popular.^7^ To our knowledge, there have been no reports of arthroscopic repair of the attachment point of the talus. Our purpose is to describe how to perform arthroscopic ligament repair of ATFL injuries at the talar attachment point.

## Surgical Technique

Surgical repair of the ATFL for a talar-side injury is shown in Video 1. Table 1 lists advantages and disadvantages of this approach, and Table 2 lists pearls and pitfalls.

**Table 1:** Advantages, Risks, and Limitations of Technique

Advantages
Compared with open surgery, the described technique is expected to cause fewer wound complications and patients can start rehabilitation earlier.
It is possible to assess the condition of intra-articular lesions and perform additional interventions.
Disadvantages
There is a risk of injury to the superficial peroneal nerve when an anterolateral portal is created.
Limitations
The technique is limited to cases with or without small bone fragments.
The technique is dependent on remnant ligament quality.

**Table 2:** Surgical Steps, Pearls, and Pitfalls

Surgical Steps	Pearls	Pitfalls
Patient positioning	The foot is placed in traction with an ankle distractor.	The lower limbs are often in an externally rotated position. Therefore, if a neutral position cannot be maintained, it is necessary to devise a way to place an airbag under the buttocks.
Marking of landmarks	The ATFL, fibula, and talus are marked using ultrasonography to confirm the location of the ligament footprint.	These markings play an important role in portal creation during surgery. Thus, care must be taken to ensure that the markings do not disappear during disinfection.
Intra-articular evaluation	The anterolateral portal is used as a viewing portal to evaluate the quality of the ATFL and the ligament attachment. Adipose tissue and synovium that obstruct vision are removed.	A shaver is mainly used, but it is necessary to use a radiofrequency ablation device near the ligaments because there is a possibility of damage.
Creation of far accessory anterolateral portal	The far accessory anterolateral portal is placed directly above the footprint on the talar side under arthroscopic, ultrasonographic, and fluoroscopic imaging.	The footprint is confirmed by multiple means, not just preoperative markings, and a fully operational portal is created.
Insertion of suture anchor	When operating the drill into the footprint, the surgeon should be conscious of drilling in the direction of the medial malleolus.	The ATFL talar attachment is at the end of the articular cartilage, so if it is placed in the wrong direction, it will cause cartilage damage.
Tightening of suture fiber	The knotless sutures are tightened, and the elongated ligament is advanced in the ankle dorsiflexion position.	Failure to maintain proper limb position can result in ligaments being loose or too tight.
Postoperative care	Plantar flexion should be restricted for 3 wk after surgery.	Because the ATFL is tensed during plantar flexion, there is a risk of rupture in the early postoperative period.

ATFL, anterior talofibular ligament.

### Preoperative Assessment

**Fig 1:**
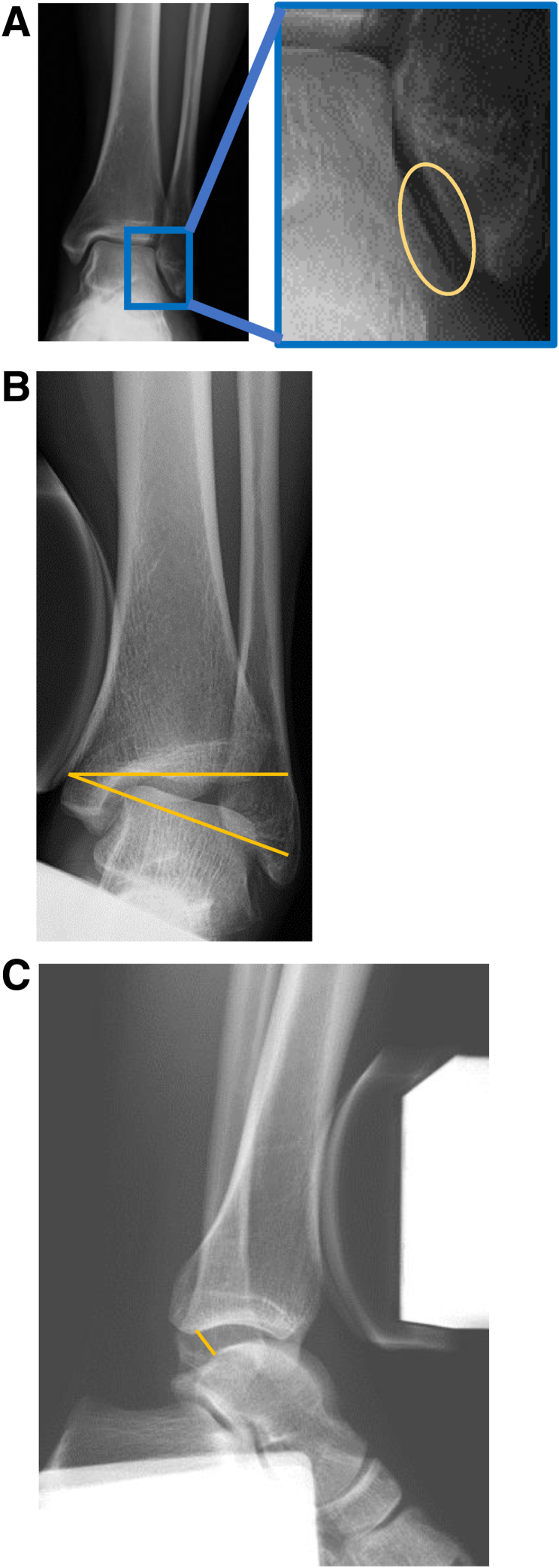
Preoperative radiographic images of left ankle: anteroposterior view. (A) A thin bone fragment is observed between the talus and fibula (oval). (B, C) Stress radiographs are obtained using a Telos stress device with a magnitude of 150 N. The inversion stress test shows an increase in the talar tilt angle (lines) (B), and the anterior drawer test shows an increase in the anterior drawer distance (line) (C).

**Fig 2:**
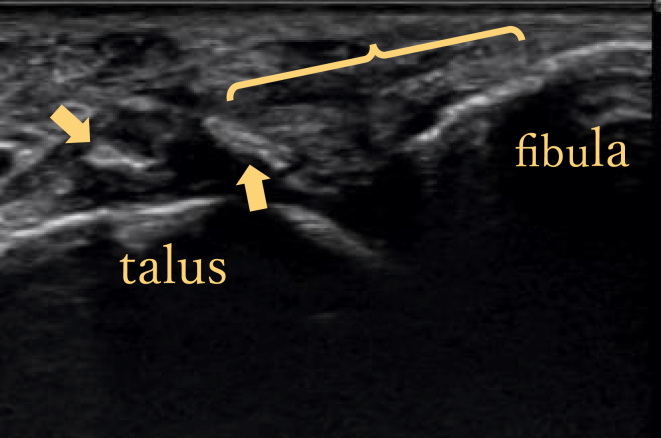
Ultrasonographic findings of anterior talofibular ligament injury site: detached talar bone fragment (arrows) and deflection of anterior talofibular ligament (bracket).

**Fig 3:**
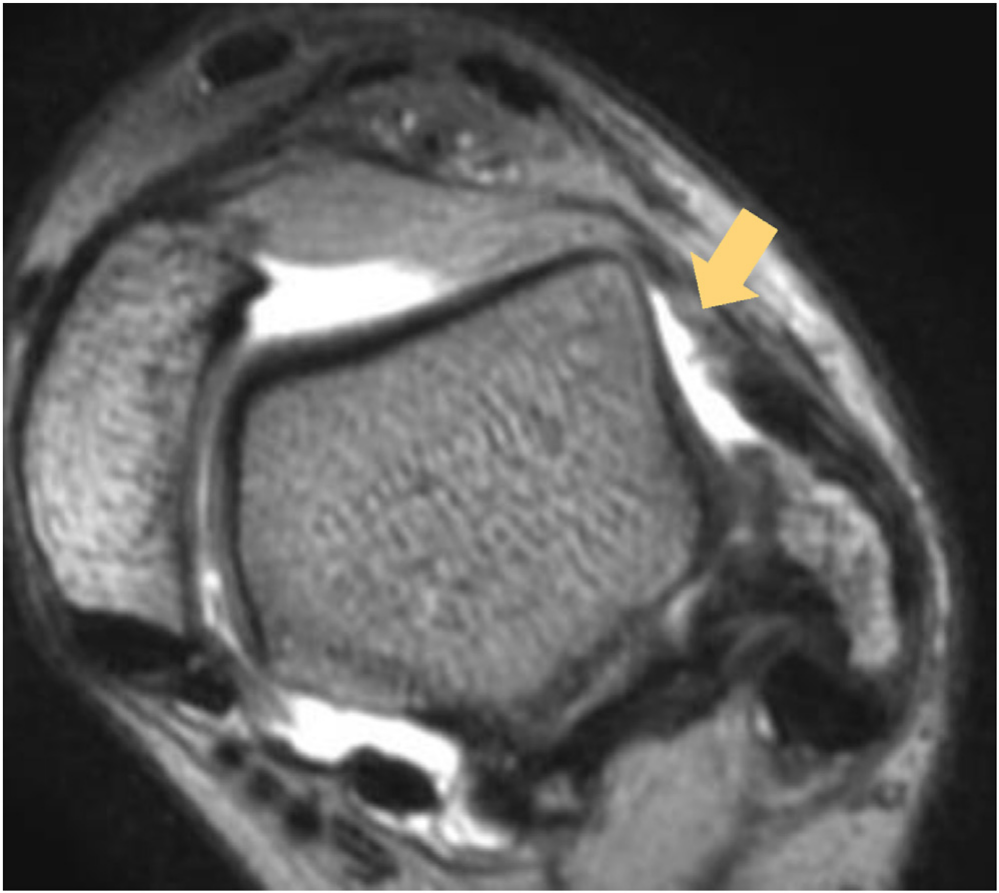
T2-weighted magnetic resonance imaging of anterior talofibular ligament (ATFL) injury site: oblique axial view. Normally, the fibers of the ATFL would appear as a bundle of low-intensity areas, but this image shows irregularity of the ATFL and detachment on the talar side (arrow).

A common cause of ATFL injury is ankle inversion, and a detailed medical history must be taken to understand the classification and mechanism of injury. It is also important to evaluate the point of tenderness by palpation. Imaging tests include radiographs, ultrasonography, and magnetic resonance imaging. Radiographs of the ankle are primarily used to evaluate bony abnormalities such as avulsion fractures (Fig 1A). Inversion stress radiography using a Telos stress device (Griesheim, Germany) with a magnitude of 150 N shows a greater talar tilt angle (Fig 1B) and anterior drawer distance (Fig 1C) compared with the healthy side. Ultrasonography can noninvasively evaluate ligaments and bone fragments (Fig 2), whereas magnetic resonance imaging can be used to assess ligament integrity (Fig 3).

### Patient Positioning

The patient is placed in the supine position. Surgery is performed with the patient under general and conductive anesthesia using a tourniquet under joint distraction with an ankle distractor (Spider Limb Positioner; Smith & Nephew, Watford, England). If the hip joint tends to be in external rotation, cushioning material is placed under the buttocks of the affected side and the patient is placed in a semi-lateral position.

### Arthroscopic Findings

**Fig 4:**
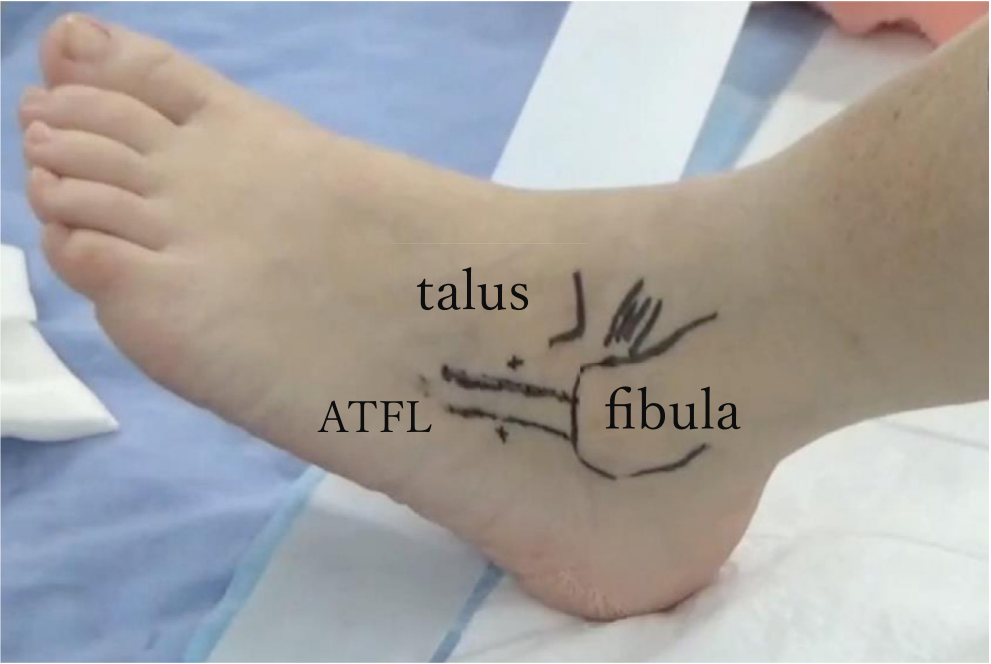
Patient's left leg landmarks showing anterior talofibular ligament (ATFL), fibula, and talus created using ultrasonography. The course of the ATFL is continuous with the fibula but is separated on the talar side and runs laterally. The far accessory anterolateral portal is marked distal to the fibula and on the talar side of the ATFL.

**Fig 5:**
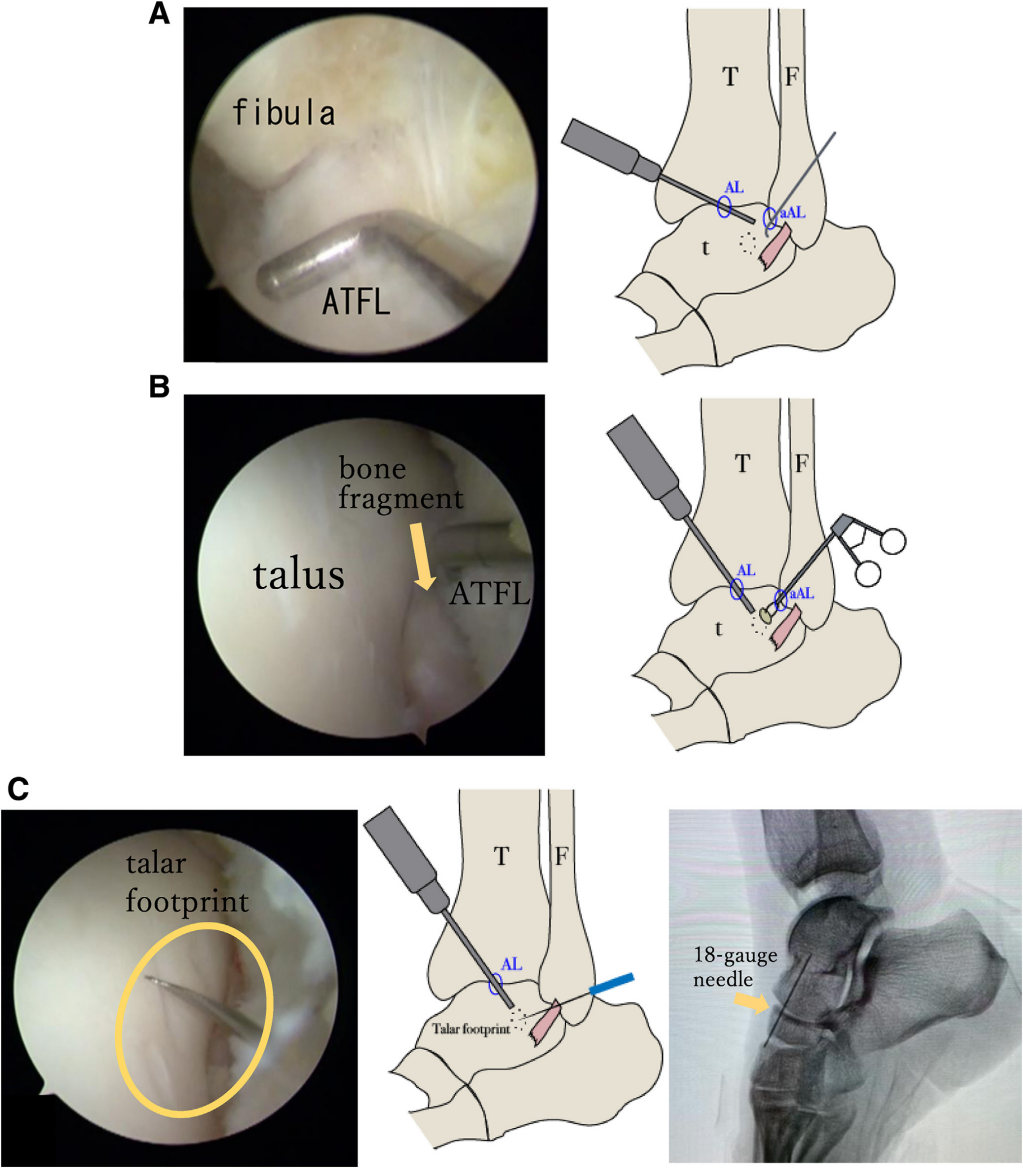

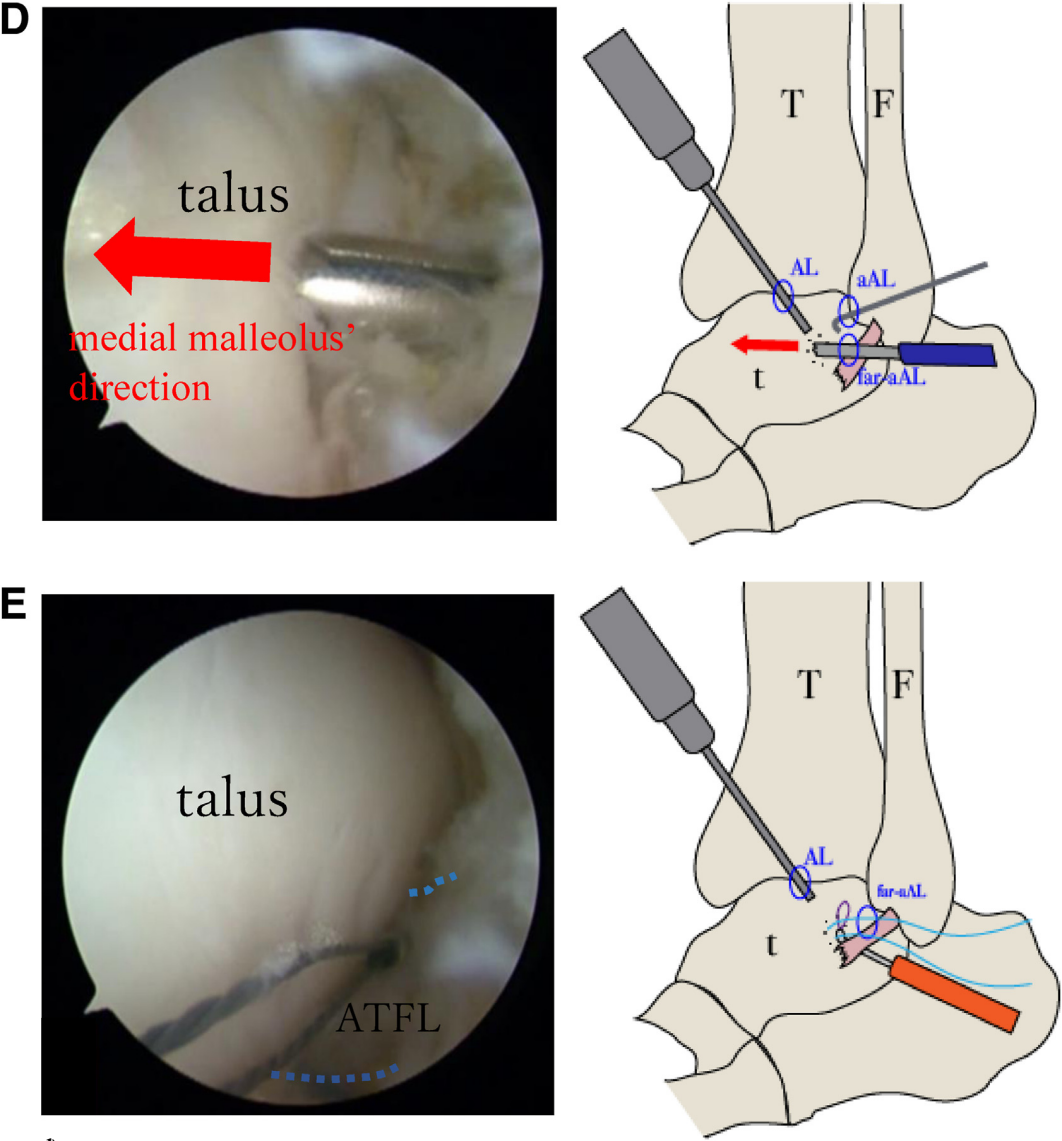

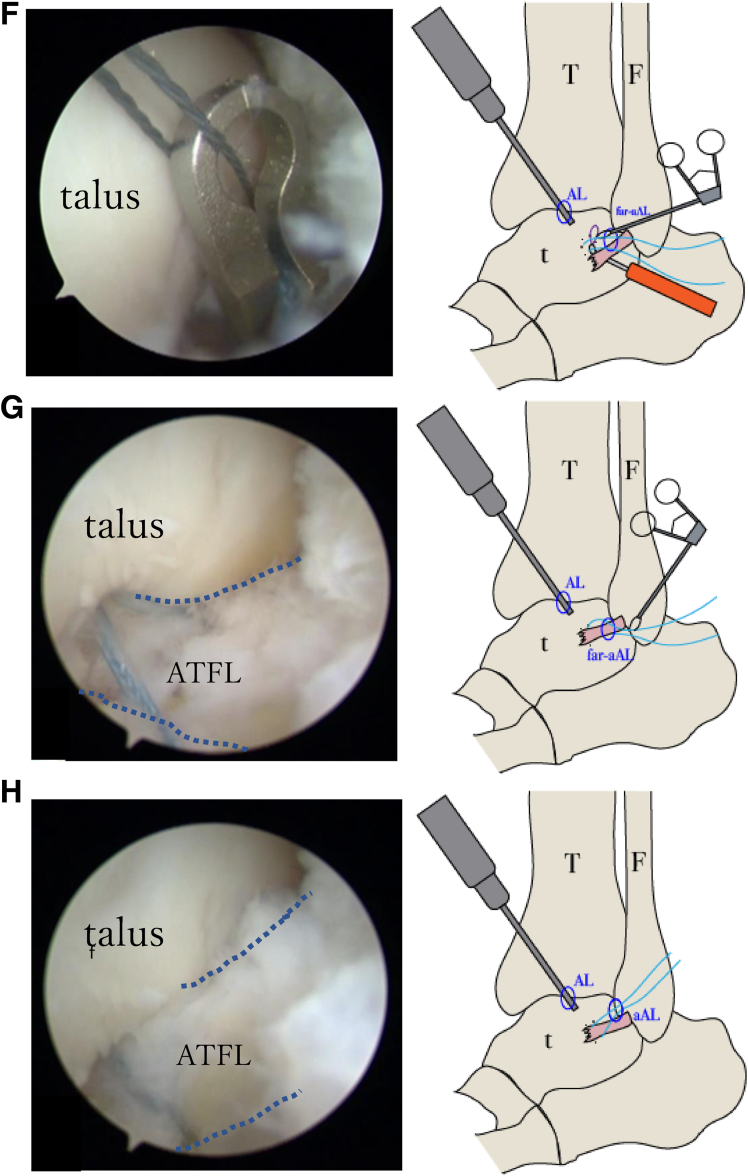
Intraoperative findings. The accessory anterolateral (aAL) portal is created as a working portal, and the anterior talofibular ligament (ATFL) is evaluated using the anterolateral (AL) portal as the viewing portal. (A) Assessment of ligament quality using a probe reveals no damage or degeneration of the ATFL in the fibular region adjacent to the ligament attachment. (B) When viewed from the AL portal, the ATFL is torn on the talar side, accompanied by small bone fragments (yellow arrow). Bone fragments are removed from the aAL portal. (C) On the basis of the markings created using ultrasonography, the footprint (yellow oval) on the talar side is confirmed under imaging using an 18-gauge needle using the AL portal as the viewing portal, and a far a AL portal is placed directly above the footprint of the talar ligament attachment.. (D) We drill into the talar footprint in the direction of the medial malleolus (red arrows) using the AL portal as the viewing portal and insert a suture anchor at the talar insertion of the ATFL through the far aAL portal. (E) The ATFL (blue dotted lines) is lifted with a needle, and a guidewire is inserted from the far aAL portal across the ATFL using the AL portal as the viewing portal. (F) The anchor thread is passed through the guidewire loop, and sutures are passed through the ligament from the far aAL portal. (G, H) The threads are concentrated in the aAL portal and sutured in the dorsiflexion position. The ATFL (blue dotted lines) is fixed without loosening. (F, fibula; t, talus; T, tibia.)

The ATFL, fibula, and talus are marked using ultrasonography to confirm the location of the ligament footprint. Meanwhile, the surgeon should mark the footprint of the ATFL on the talar side for the creation of the far accessory anterolateral portal (Fig 4). A 2.7-mm, 30° arthroscope (Dyonics; Smith & Nephew) is used for this procedure. The anteromedial portal is created just medial to the anterior tibial tendon in the joint space. The anterolateral portal is made using a needle just lateral to the peroneus tertius under arthroscopic guidance from the anteromedial portal. First, the quality of the articular cartilage and the severity of synovitis are evaluated using the anteromedial portal as a viewing portal. To evaluate the quality of the ATFL, the accessory anterolateral portal is used for working and the anterolateral portal is used for viewing. The accessory anterolateral portal is created distal to the lateral malleolus and proximal to the ATFL marking. Adipose tissue and synovium are removed for visibility using a shaver (Dyonics Power Max; Smith & Nephew) and a radiofrequency ablation device (Vulcan radiofrequency ablation system; Smith & Nephew). Assessment of ligament quality using a probe reveals no damage or degeneration of the ATFL in the fibular region adjacent to the ligament attachment (Fig 5A). ATFL injuries are present at the talar footprint, and the ligament tissue is attenuated with small bone fragments.

### Procedure

The far accessory anterolateral portal is needed to approach the talar footprint and is placed directly above the footprint on the talar side under arthroscopic, ultrasonographic, and fluoroscopic imaging (Fig 5B). The bony bed of the anatomic footprint of the talus is refreshed using a shaver and a radiofrequency ablation device after the bone fragments are removed (Fig 5C).

Subsequently, a suture anchor guide is inserted through the far accessory anterolateral portal, followed by drilling into the talar footprint in the direction of the medial malleolus, and a suture anchor (DEX Knotless FiberTak anchor; Arthrex, Naples, FL) is inserted at the talar insertion of the ATFL (Fig 5D). A suture fiber is passed through the entire ligament using a suture passer (Micro SutureLasso; Arthrex) that penetrates beneath the ligament (Fig 5E-G). While the ATFL is lifted, the knotless sutures are tightened and the elongated ligament is advanced in the ankle dorsiflexion position (Fig 5H). Finally, the presence of stability during the anterior drawer test is confirmed, and the absence of suture loosening is verified under arthroscopic visualization. Four small incisions are closed using a nonabsorbable No. 4-0 suture without a drain.

### Postoperative Care

Immediately after surgery, the patient is placed in an ankle brace and starts a physical therapy program that includes ankle range of motion and strengthening. Ankle brace–assisted walking should start a few days after surgery, but plantar flexion is restricted for 3 weeks after surgery.

## Discussion

Chronic ankle instability due to ATFL injuries often occurs on the fibular side of the ankle but is occasionally observed on the talar side. The location of the talar footprint of the ligament is confirmed using fluoroscopy, ultrasound imaging, and arthroscopy. The suture anchor is then inserted into the anatomic enthesis, as in the arthroscopic repair technique at the fibular site. We have found that repair of an ATFL injury on the talar side is possible under arthroscopy, and good results are obtained. Arthroscopic repair may be preferred for ATFL injuries on the talar side.

ATFL injuries tend to occur on the fibular side because the ligament is exposed to greater tension on the fibular side than on the talar side. Talar injuries have rarely been reported in ATFL injuries involving avulsion fractures. Kumai et al.^8^ reported that the attachment on the talar side had a higher bone density than that on the fibular side. Additionally, the properties of the fibrocartilage near the talar articular surface disperse stress, making avulsion fractures less likely to occur on the talar side than on the fibular side.^8^

Using an open repair method, Broström^5^ reported an anatomic repair technique that involved suturing the ATFL and calcaneofibular ligament, along with the joint capsule. The Broström method can be further enhanced by incorporating the extensor retinaculum for repair.^9^ Currently, these modified arthroscopic techniques have become the gold standard.^10^ Rigby and Cottom^11^ compared the open modified Broström-Gould procedure with the all-inside arthroscopic procedure and suggested the potential benefits of the arthroscopic approach, citing fewer wound complications, the possibility of early weight bearing, and the ability to perform additional interventions for intra-articular lesions.

In arthroscopic ATFL repair for avulsion fractures, small bone fragments from the fibula or talus can lead to complications and, if left behind, may become a significant source of pain.^12^ Therefore, bone fixation is generally preferred for larger fragments and excision, for smaller fragments. In this case, we removed the bone fragments because of the possibility that leaving the small bone fragments could cause residual irritating pain. In a study by Ozkan et al.^13^ that about cases of recent injury involving large bones, open fixation was performed using a cannulated headless compression screw.

Arthroscopic repair of the ATFL is associated with the risk of neurologic complications. The far accessory anterolateral portal is located in the safe zone in the lateral ankle region.^14^ After the skin incision, the subcutaneous tissue is sufficiently separated, and no postoperative neurologic complications have been observed. A limitation of this technique is that it is restricted to cases in which the talar bone fragment is small.

Arthroscopic all-inside ligament repair has the potential to facilitate early postoperative mobilization. Even on the talar side, arthroscopic repair using ultrasound and fluoroscopic imaging may be safe and minimally invasive.

## Disclosures

All authors (N.U., Y.M., K.T., Y.M., K.W., M.E., A.T.) declare that they have no known competing financial interests or personal relationships that could have appeared to influence the work reported in this paper.

## Supplementary Data

Video 1 Surgical repair of anterior talofibular ligament (ATFL) for talar-side injury. The patient is placed in the supine position. A tourniquet is used, and counter support is attached to the thigh under joint distraction with an ankle distractor (Spider Limb Positioner). Preoperatively, ultrasonography reveals a detached talar bone fragment and ATFL deflection. The ATFL, fibula, and talus are marked using ultrasonography to confirm the location of the ligament footprint. The surgeon marks the location of the far accessory anterolateral portal on the talar footprint of the ATFL. A 2.7-mm, 30° arthroscope is used. The anteromedial portal and anterolateral portals are created for viewing and working, respectively. The anteromedial portal is created just medial to the anterior tibial tendon, palpating the joint space. Arthroscopy is performed through the anteromedial portal, and the anterolateral portal is created by inserting a needle just lateral to the peroneus tertius. There is no articular cartilage injury or degeneration. To evaluate the quality of the ligament at the fibular attachment, an accessory anterolateral portal is created distal to the lateral malleolus and proximal to the ATFL marking as the viewing portal. Soft tissue and synovium are removed for visibility using a shaver and a radiofrequency ablation system. Evaluation of ligament quality using a probe reveals no ATFL injury or degeneration in the fibular region adjacent to the ligament attachment. On evaluation of the talar side viewing from AL portal, ATFL tears are present at the talar footprint,and the ligament tissue is attenuated with small bone fragments, which are removed. The soft tissue attached to the bone fragment is removed using a radiofrequency ablation device to avoid damaging the ATFL. The following operations used the AL portal as the viewing portal. The far accessory anterolateral portal is placed directly above the talar footprint under arthroscopic, ultrasonographic, and fluoroscopic imaging. On the basis of the markings made during ultrasound examination, an 18-gauge needle is inserted while the footprint on the talar side is checked under imaging, and the far accessory anterolateral portal is placed directly above the footprint at the talar attachment. A suture anchor guide is inserted through the far accessory anterolateral portal, followed by drilling into the talar footprint in the direction of the medial malleolus, and a suture anchor is placed at the talar insertion of the ATFL. If visibility is poor when the anchor is inserted, a probe is used from the accessory anterolateral portal to assist. The ATFL is enclosed with a needle, and a guidewire is inserted from the far accessory anterolateral portal across the ATFL. The anchor thread is passed through the guidewire loop, and sutures are passed through the ligament from the far accessory anterolateral portal. A suture fiber is passed through the entire ligament using a suture passer that penetrates beneath the ligament. While the ATFL is being lifted, knotless sutures are tightened and the elongated ligament is advanced in the ankle dorsiflexion position. Finally, the sutures are confirmed not to be loose under arthroscopic vision, and the operation is completed.
